# Chronic meningitis in adults: a comparison between neurotuberculosis and neurobrucellosis

**DOI:** 10.1186/s12879-024-09345-6

**Published:** 2024-04-25

**Authors:** Matin Shirazinia, Fereshte Sheybani, HamidReza Naderi, Mahboubeh Haddad, Pouria Hajipour, Farzaneh Khoroushi

**Affiliations:** 1 Faculty of Medicine, Mashhad University of Medical Sciences, Mashhad, Iran; 2 Department of Infectious Diseases and Tropical Medicine, Imam Reza Teaching Hospital, Faculty of Medicine, Mashhad University of Medical Sciences, Daneshgah Street, Mashhad, Iran; 3 Department of Radiology, Faculty of Medicine, Mashhad University of Medical Sciences, Mashhad, Iran

**Keywords:** Subacute meningitis, Chronic meningitis, Neurobrucellosis, Neurotuberculosis, Brucellosis, Tuberculosis

## Abstract

**Background:**

In regions endemic for tuberculosis and brucellosis, distinguishing between tuberculous meningitis (TBM) and brucella meningitis (BM) poses a substantial challenge. This study investigates the clinical and paraclinical characteristics of patients with TBM and BM.

**Methods:**

Adult patients diagnosed with either TBM or BM who were admitted to two referral hospitals between March 2015 and October 2022, were included, and the characteristics of the patients were analyzed.

**Results:**

Seventy patients formed the study group, 28 with TBM and 42 with BM, were included. TBM patients had a 2.06-fold (95% CI: 1.26 to 3.37, *P*-value: 0.003) higher risk of altered consciousness and a 4.80-fold (95% CI: 1.98 to 11.61, *P*-value: < 0.001) higher risk of extra-neural involvement as compared to BM patients. Cerebrospinal fluid (CSF) analysis revealed a significantly higher percentage of polymorphonuclear leukocytes (PMN) in TBM compared to BM (Standardized mean difference: 0.69, 95% CI: 0.18 to 1.20, *P*-value: 0.008). Neuroimaging findings indicated higher risks of hydrocephalus (*P*-value: 0.002), infarction (*P*-value: 0.029), and meningeal enhancement (*P*-value: 0.012) in TBM compared to BM. Moreover, TBM patients had a 67% (95% CI: 21% to 131%, *P*-value:0.002) longer median length of hospital stay and a significantly higher risk of unfavorable outcomes (Risk ratio: 6.96, 95% CI: 2.65 to 18.26, *p* < 0.001).

**Conclusions:**

Our study emphasizes that TBM patients displayed increased frequencies of altered consciousness, PMN dominance in CSF, extra-neural involvement, hydrocephalus, meningeal enhancement, and brain infarction. The findings emphasize the diagnostic difficulties and underscore the importance of cautious differentiation between these two conditions to guide appropriate treatment strategies.

**Supplementary Information:**

The online version contains supplementary material available at 10.1186/s12879-024-09345-6.

## Introduction

Subacute or chronic meningitis refers to inflammation of the meninges that develops over days to weeks [1]. It is a serious category of neurological infections and is associated with significant morbidity and mortality. Given the wide range of potentially infectious and non-infectious causes, subacute and chronic meningitis are diagnostically challenging [2]. Over the last decades, using molecular analysis, new infectious agents have been included in the existing list of etiological agents of subacute and chronic meningitis [3]. Globally, the two major causes of subacute and chronic meningitis are cryptococcal meningitis and tuberculous meningitis (TBM) [4, 5]. In addition, in countries such as Iran where brucellosis is endemic, brucella meningitis (BM) is another major cause of subacute and chronic meningitis [6, 7].

In the regions that are endemic for both tuberculosis and brucellosis, one of the main challenges in the cause-specific diagnosis of chronic meningitis, is the differentiation between meningitis caused by *Mycobacterium tuberculosis* and Brucella sp. [8]. Because of the overlapping manifestations of the neurological syndromes caused by *M. tuberculosis* and Brucella sp., making a distinction between them is challenging [9]. Moreover, microbiological tests have low yield in isolating the causative pathogens of these two causes of chronic meningitis [9, 10]. Here, we report the characteristics of patients with TBM and BM in Mashhad, Iran and compare their clinical, laboratory, and imaging characteristics.

## Methods

### Study design and setting

Between March 2015 and October 2022, all adults (15 years of age or older) diagnosed with TBM or BM who were hospitalized in the two main referral hospitals for neuroinfections and neuroinflammations in Mashhad, Iran, were enrolled. Mashhad, the capital of Razavi Khorasan province, is in the northeastern part of Iran and has the second-highest population among Iranian cities [11].

Between March 2015 and September 2019, we conducted a retrospective review of medical records and discharge letters for admitted patients. For individuals admitted between October 2019 and October 2022, the data were prospectively recorded through an online patient registration system for community-acquired brain infections and inflammations, and the data of included patients were collected from this registration system.

### Inclusion and exclusion criteria

Brucellosis was identified through clinical signs and symptoms suggestive of the disease, accompanied by the detection of Brucella sp. either in blood culture or through the presence of antibodies against brucella in the serum. Throughout the duration of the research, traditional culture media (incubated for 2–3 weeks) were utilized for cerebrospinal fluid (CSF) culture, while standard BACTEC™ Plus Aerobic/F bottles (incubated for one week) were employed for blood cultures. According to the Iranian national protocol for diagnosing and managing brucellosis in endemic regions, the diagnosis of brucellosis was made based on clinical findings and on either positive culture for Brucella sp. or the presence of serum antibodies (standard tube agglutination (STA) test titer ≥ 1/80, Coombs test titer ≥ 1/40, and 2-Mercaptoethanol (2-ME) test titer ≥ 1/40) [12–14]. These cut-offs took into account the exclusion of other potential differential diagnoses. Regarding CSF, the threshold was set at titer of 1/8 in the STA test. The diagnosis of BM in individuals with confirmed brucellosis was established by the presence of at least one of the following: (I) suspected signs and symptoms of neurobrucellosis, including confusion, a persistent and severe headache, depression, changes in behavior, incontinence, insomnia, nuchal rigidity, and any neurological abnormalities discovered during evaluation; (II) detection of Brucella sp. in CSF and/or the presence of positive anti-brucella antibodies in CSF; (III) increased protein levels, reduced glucose levels, or identification of pleocytosis in CSF; or (IV) observation of abnormal findings in cranial magnetic resonance imaging (MRI) or computed tomography (CT) scans [15].

As patients with BM may be missclassified as either possible or probable cases of TBM, we exclusively considered definite cases of TBM as defined by the study of Marais et al. [16] and the presumptive cases were excluded.

### Data collection and measurements

Patients’ demographic data, underlying comorbidities, clinical manifestations, laboratory tests, imaging, and clinical outcomes were recorded. Regarding CSF analysis, only the initial CSF sample was reported. Mild pleocytosis was defined as the presence of fewer than 50 white blood cells (WBC) per microlitre in the CSF sample. Severe hyperproteinorrhachia was characterized by a protein amount ≥ 500 mg/dL, and severe hypoglycorrhachia was defined as glucose levels < 10 mg/dL in the CSF specimen.

Moreover, the picture archiving and communication system (PACS) of the participating hospitals was used for the evaluation of CT scans and MRI of the patients. To ensure the reliability and validity of radiologic assessments, an experienced radiologist evaluated all images. The outcome was scored according to the Glascow outcome scale (GOS) [17]. A favorable outcome was defined as a score of 5, and an unfavorable outcome was defined as a score of 1 to 4.

### Statistical analysis

Statistical analyses were conducted with STATA version 14.2 (Stata, College, Statin, Texas) and R version 4.3.1 (R development core team, University of Auckland, New Zealand). Normality was evaluated using Shapiro–wilk test and P-P plot. For baseline characteristics, continuous data were described with the median and interquartile range (percentile 25 to percentile 75) and categorical variables with the frequency and percentage. The Mann–Whitney U-test were used for continuous variables, while Fisher exact test and the Chi-square test were used for categorical variables, as appropriate.

Regarding CSF parameters, univariable linear regression analysis was used to evaluate differences between TBM and BM. The assumption of normality of residuals was checked through P-P plots and the Shapiro–Wilk test. As this assumption was violated across all CSF parameters, appropriate transformations were considered. A log transformation was applied to CSF protein and glucose, with the results subsequently presented using the exponential function as the geometric mean. The effect size was indicated as the ratio of geometric mean (RoGM). For the total WBC count and the percentage of polymorphonuclear (PMN) in the CSF sample, a square root transformation was applied. The mean was reported after back transformation, and the effect size was presented as the β coefficient in the scale of square root. Using the *esizereg* [18] post-estimation command in STATA, the β coefficient was subsequently converted to the standardized mean difference (SMD). Furthermore, the assumption of homoscedasticity was examined using *rvfplot* and the *estat hettest* post-estimation command in STATA. Additionally, the impact of leverage (within an acceptable range of less than 3*(k + 1)/n) and outliers (within an acceptable range of -3.29 to 3.29) was assessed.

The clinical and paraclinical presentations were compared, and the risk ratio (RR) along with its corresponding 95% confidence interval was reported. Additionally, parametric survival models (using Weibull, exponential, log-normal, and log-logistic distributions) were utilized to compare the median length of hospital stay (LOS) using the time ratio (TR). The appropriate model was chosen based on the preference of Akaike information criterion (AIC) and Bayesian information criterion (BIC). In all analysis, a *P* < 0.05 was considered statistically significant.

## Results

### Baseline characteristics

A total of 70 patients were included in the study, of whom 28 had TBM, and 42 had BM. The median age of patients with TBM and BM was 39.5 [24.5 to 55.5] and 31 [24 to 46] years, respectively (Table 1). The most prevalent comorbidity was cardiovascular disorders in both the TBM (*N* = 2, 7.4%) and BM (*N* = 3, 7.3%) groups.

**Table 1:** Baseline characteristics of patients with tuberculous meningitis and Brucella meningitis

	**TBM** **(*n* = 28)**	**BM** **(*n* = 42)**	***P*-value**
Age (years), median (percentile 25 to percentile 75)	39.5 (24.5 to 55.5)	31 (24 to 46)	0.193
Elderly^a^, n (%)	6 (21.4)	4 (9.5)	0.183
Sex (male), n (%)	13 (46.4)	27 (64.3)	0.139
Underlying comorbidity, n (%)			
Cardiovascular disorders	2/27 (7.4)	3/41 (7.3)	1.000
Diabetes mellitus	2/27 (7.4)	0/41	0.154
Dementia	1/26 (3.9)	0/41	0.388
HIV/AIDS	1/27 (3.7)	0/41	0.397
Rheumatologic disorders	1/27 (3.7)	0/41	0.397
Stroke	0/26	1/41 (2.4)	1.000

*TBM* Tuberculous meningitis, *BM* Brucella meningitis, *HIV* Human immunodeficiency virus, *AIDS* acquired immunodeficiency syndrome

^a^Defined as age of ≥ 65 years-old

### Clinical, laboratory, and imaging characteristics

TBM, when compared with BM, showed a significant 2.06-fold increase in the risk of altered consciousness (RR: 2.06, 95% CI: 1.26 to 3.37, *P*-value: 0.003) (Table 2). The risk of extra-neural involvement was significantly higher in TBM compared to BM (RR: 4.80, 95% CI: 1.98 to 11.61, *P*-value: < 0.001).

**Table 2:** Clinical presentations of patients with tuberculous meningitis and Brucella meningitis

	**TBM (*n* = 28)**	**BM (*n* = 42)**	**Risk ratio (95% CI)**	***P*-value**
Headache, n (%)	15/21 (71.4)	37/40 (92.5)	0.77 (0.58 to 1.03)	0.052
Nausea/Vomiting, n (%)	11/20 (55.0)	26/35 (74.3)	0.74 (0.48 to 1.15)	0.143
Fever, n (%)	19/21 (90.5)	29/38 (76.3)	1.19 (0.95 to 1.49)	0.297
Altered consciousness^a^, n (%)	19/27 (70.4)	14/41 (34.2)	2.06 (1.26 to 3.37)	**0.003**
Seizures, n (%)	4/26 (15.4)	6/40 (15.0)	1.03 (0.32 to 3.29)	1.000
Neck stiffness, n (%)	5/19 (26.3)	13/30 (43.3)	0.61 (0.26 to 1.43)	0.229
Neurologic deficits, n (%)	9/20 (45.0)	8/36 (22.2)	2.03 (0.93 to 4.42)	0.076
Extra-neural involvement, n (%)	16 (57.1)	5 (11.9)	4.80 (1.98 to 11.61)	** < 0.001**

*TBM* Tuberculous meningitis, *BM* Brucella meningitis, *95% CI* 95% confidence interval

^a^Defined as Glasgow coma scale (GCS) of < 15

**Fig. 1:**
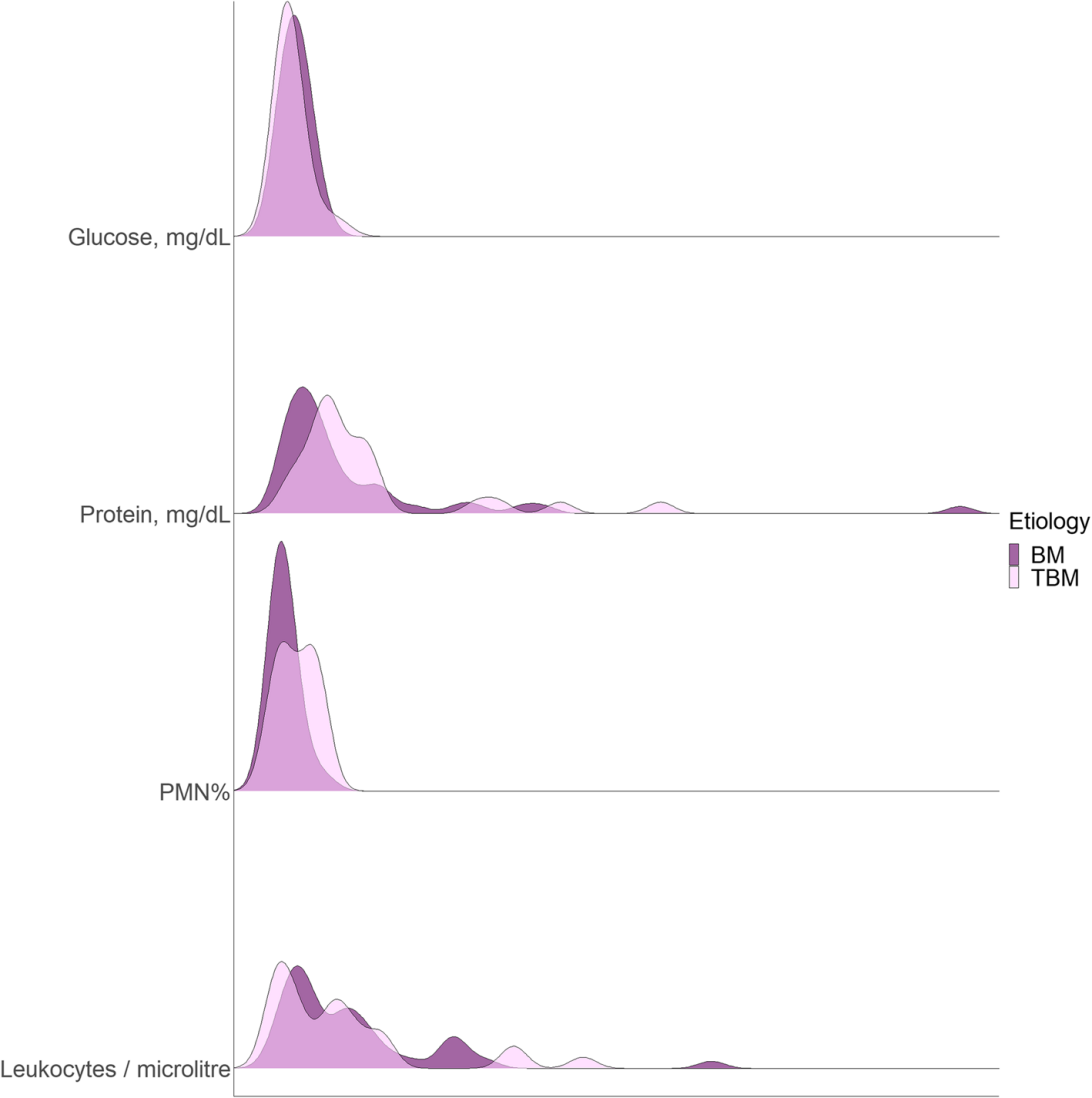
Distribution of cerebrospinal fluid indices between two groups of tuberculous meningitis and brucella meningitis. TBM, tuberculous meningitis; BM, brucella meningitis; CSF, cerebrospinal fluid; mg, milligram; dL, decilitre; PMN, polymorphonuclear leukocytes

The distribution of CSF parameters in two groups was shown in Fig. 1. The CSF analysis revealed that the PMN percentage in TBM was significantly higher than in BM (SMD: 0.69, 95% CI: 0.18 to 1.20, *P*-value: 0.008) (Table 3). The probability of PMN dominance in CSF was significantly greater in TBM compared to BM (RR: 4.62, 95% CI: 1.67 to 12.78, *P*-value: 0.001). Moreover, there was a marginally significant 52% increase in the geometric mean of CSF protein in TBM compared to BM (RoGM: 1.52, 95% CI: 0.96 to 2.41, *P*-value: 0.076). All assumptions and concerns of linear regression were met in the analyses.

**Table 3:** Cerebrospinal fluid analysis and Neuroimaging findings of patients with tuberculous meningitis and Brucella meningitis

		**TBM (*n* = 28)**	**BM (*n* = 42)**	**Effect size (95% CI)**	***P*-value**
CSF analysis	CSF leukocytes/ microlitre, mean^a^ (95% CI)	86.0 (45.1 to 139.9)	116.2 (81.6 to 157.1)	β coefficient^b^: -1.51 (-4.44 to 1.43)	0.308
	Mild pleocytosis (leukocytes < 50/ microlitre), n (%)	12/27 (44.4)	11/42 (26.2)	RR: 1.70 (0.88 to 3.28)	0.116
	CSF PMN percentage^a^, mean (95% CI)	34.1 (20.6 to 50.9)	16.1 (10.9 to 22.4)	β coefficient^b^: 1.82 (0.48 to 3.16)	**0.008**
	CSF PMN predominance, n (%)	12/26 (46.2)	4/40 (10.0)	RR: 4.62 (1.67 to 12.78)	**0.001**
	CSF protein (mg/dL), geometric mean (95% CI)	128.0 (93.4 to 175.4)	84.3 (61.7 to 115.3)	RoGM: 1.52 (0.96 to 2.41)	0.076
	Severe hyperproteinorrhachia (protein ≥ 500 mg/dL), n (%)	2/26 (7.7)	2 (4.8)	RR: 1.62 (0.24 to 10.78)	0.633
	CSF glucose (mg/dL), geometric mean (95% CI)	26.8 (20.1 to 35.7)	35.0 (28.8 to 42.6)	RoGM: 0.76 (0.55 to 1.06)	0.107
	Severe hypoglycorrhachia (glucose < 10 mg/dL), n (%)	4/26 (15.4)	2 (4.8)	RR: 3.23 (0.64 to 16.41)	0.193
Neuroimaging findings	Hydrocephalus, n (%)	10/27 (37.0)	2/40 (5.0)	RR: 7.41 (1.76 to 31.19)	**0.002**
	Infarct, n (%)	7/27 (25.9)	2/37 (5.4)	RR: 4.80 (1.08 to 21.31)	**0.029**
	Granuloma/Abscess, n (%)	5/26 (19.2)	3/34 (8.8)	RR: 2.18 (0.57 to 8.30)	0.275
	Meningeal enhancement, n (%)	8/25 (32.0)	2/35 (5.7)	RR: 5.60 (1.30 to 24.16)	**0.012**
	Nonspecific white matter lesions on T2-weighted and FLAIR images, n (%)	3/19 (15.8)	10/26 (38.5)	RR: 0.41 (0.13 to 1.29)	0.097

*TBM* Tuberculous meningitis, *BM* Brucella meningitis, *95% CI* 95% confidence interval, *CSF* cerebrospinal fluid, *mg* milligram, *dL* deciliter, *PMN* Polymorphonuclear leukocytes, *RoGM* Ratio of geometric mean, *RR* Risk ratio

^a^Means were computed following back-transformation from the square root transformation

^b^Coefficients were expressed in the square root scale

**Fig. 2:**
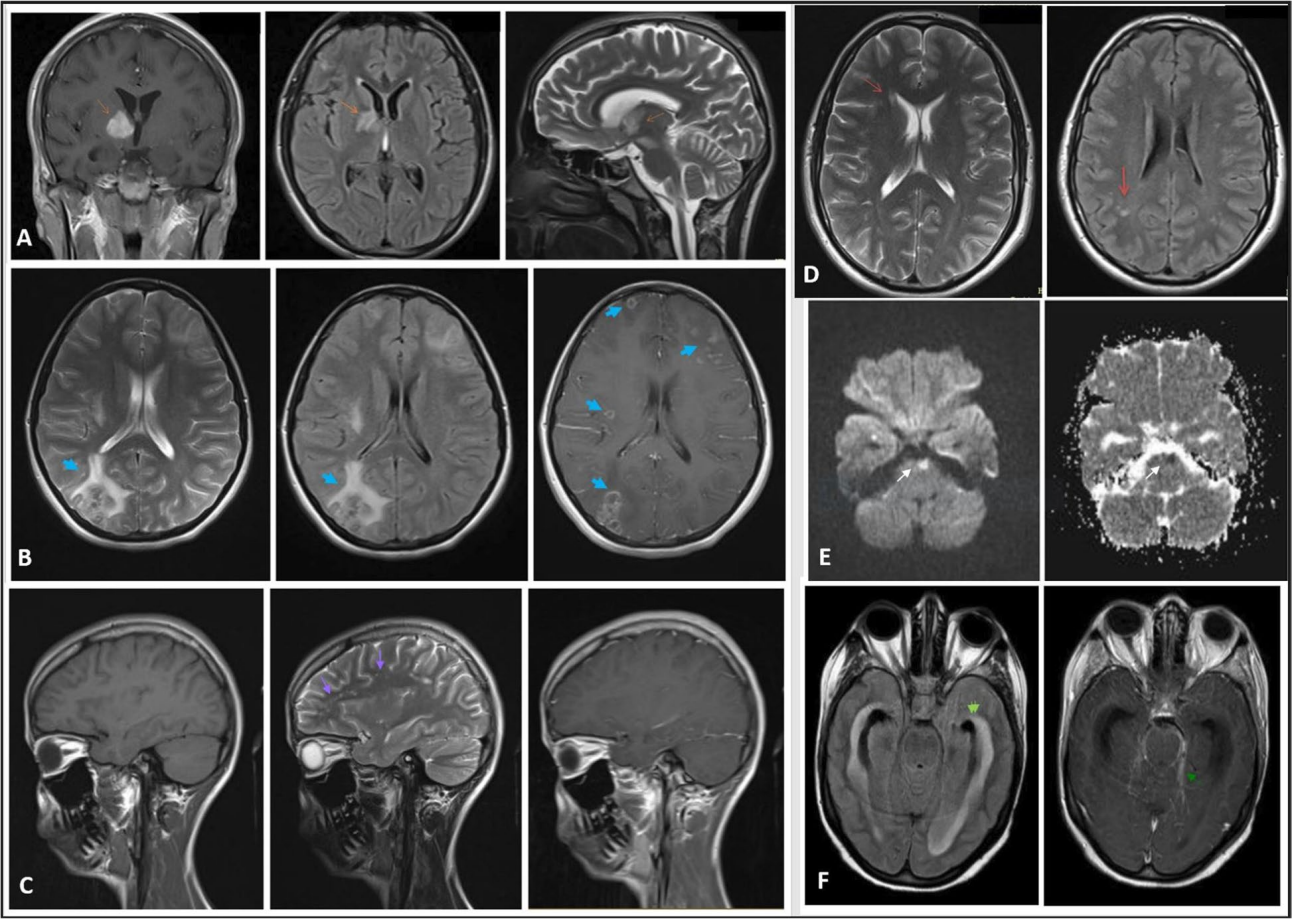
Neuroimaging findings of patients with neurotuberculosis or neurobrucellosis. Brain MRI of a 19-year-old man with neurobrucellosis presented with multiple cranial nerve palsies, papilledema, and left hemiparesis. Coronal T1/postcontrast image shows enhancing lesion in right basal ganglia, axial FLAIR image shows high signal intensity in right basal ganglia, and sagittal T2 weighted image shows high signal intensity in right basal ganglia and cerebral peduncle (orange arrows) (**A**). Brain MRI of a 16-year-old girl with TB meningoencephalitis presented with headache, confusion, and multiple cranial nerve palsies. T2, FLAIR, and T1/postcontrast images show multiple ring-enhancing lesions with vasogenic edema (blue arrows) (**B**). Brain MRI of a 20-year-old woman with brucella meningitis presented with pseudotumor cerebri-like presentation. T1, FLAIR, and T1/postcontrast images show non-specific hyper signal foci without edema or enhancement in the centrum semiovale (purple arrows) (**C**). Brain MRI of a 35-year-old man with brucella meningitis. Axial T2 weighted and FLAIR images show non-enhancing small subcortical and periventricular high signal intensities (red arrows) (**D**). Brain MRI of a 34-year-old woman with TB meningoencephalitis presented with headache, altered consciousness, seizures, multiple cranial nerve palsies, and right hemiparesis. DWI (Diffusion weighted image) shows high signal lesion in left side of pons and ADC (apparent diffusion coefficient) image shows correlation between ADC and DWI image (restriction) (white arrows) (**E**). FLAIR, and T1/postcontrast images show hydrocephalus with trans ependymal edema and leptomeningeal enhancement (green arrows) (**F**)

Neuroimaging findings suggest that the risks of hydrocephalus (RR: 7.41, 95% CI: 1.76 to 31.19, *P*-value: 0.002), infarction (RR: 4.80, 95% CI: 1.08 to 21.31, *P*-value: 0.029), and meningeal enhancement (RR: 5.60, 95% CI: 1.30 to 24.16, *P*-value: 0.012) were significantly higher in TBM compared to BM (Fig. 2).

### Length of hospital stay and clinical outcomes

The log-logistic distribution was chosen to compare the median LOS between two groups (Supplemental Table 1). The median LOS was 67% higher in TBM in comparison to BM (median LOS in TBM: 22 days [15 to 39], median LOS in BM: 13 days [9 to 21], TR: 1.67, 95% CI: 1.21 to 2.31, *P*-value: 0.002). Regarding the outcomes of TBM patients, seven (25.0%) individuals died, and among those who survived to hospital discharge, 13 (61.9%) experienced unfavorable outcomes. One BM patient left the hospital against medical advice, leading to uncertainty about the clinical outcome of this individual. Of other BM cases, all patients survived, with four (9.8%) of them experiencing unfavorable outcomes. The rate of death was significantly higher in TBM compared to BM (*P*-value: 0.001). TBM was associated with a higher risk of an unfavorable outcome compared to BM (RR: 6.96, 95% CI: 2.65 to 18.26, *P*-value: < 0.001).

## Discussion

In endemic areas, differentiation between TBM and BM is challenging [7]. On the one hand, their clinical manifestations often overlap, and, on the other hand, the microbiological diagnosis of these two clinical entities continues to be a challenge. Prediction systems such as Thwaites and Lancet have been developed, offering a promising avenue for the rapid diagnosis of TBM. However, these prediction systems are susceptible to misdiagnosing BM as TBM [9]. Making the diagnosis of a laboratory-confirmed BM can also be challenging because serological testing sometimes yields false negative results, and the sensitivity of culture-based methods varies depending on the laboratory techniques and quantity of bacteria in the CSF [19]. Other diagnostic methods such as CSF metagenomic next-generation sequencing (mNGS) and 16s ribosomal RNA sequencing technique are rarely available in the endemic areas [20].

Our study revealed that, among numerous overlapping features, altered consciousness, extra-neural involvement, hydrocephalus, meningeal enhancement, and brain infarction on neuroimaging, as well as the predominance of polymorphonuclear leukocytes (PMN) leukocytes in the CSF, were more frequently observed in patients with TBM than in those with BM. In addition, the percentage of PMN in the CSF of TBM patients was significantly higher than in BM patients. However, these findings are non-specific and can be used only to weigh the two alternative hypothesis states. Other clinical findings, including headache, fever, meningeal irritation, seizures, and focal neurological deficits, were not distinctive features in either TBM or BM. Also, a mild pleocytosis (< 50 leukocytes/µL), severe hypoglycorrhachia (protein ≥ 500 mg/dL), and severe hyperproteinorrhachia (glucose < 10 mg/dL) were infrequently identified at comparable frequencies in the CSF specimens of both groups.

Although the presence of nonspecific white matter lesions on T2-weighted and FLAIR images were more common in patients with BM than those with TBM, the difference was not significant in the present study. Previously, three patterns of white matter changes in BM have been described, manifesting as hyperintense lesions on T2-weighted images. These include a diffuse white matter involvement affecting the arcuate fibers region, the periventricular white matter involvement, and focal white matter changes with demyelinating appearance. The white matter involvement in BM may mimic other inflammatory or infectious disease, such as multiple sclerosis and acute disseminated encephalomyelitis (ADEM) [21]. The frequently observed imaging manifestations of TBM include hydrocephalus, tuberculomas, periventricular infarcts, meningeal enhancement, and basilar exudates [22–26]. These findings align with our study, revealing that infarction foci (RR: 4.8), meningeal enhancement (RR: 5.6), and hydrocephalus (RR: 7.4) were significantly more prevalent in TBM compared to BM.

In our study, patients with TBM had a 70% increase in the median length of hospital stay (LOS) and were seven times more at risk of experiencing unfavorable outcomes (GOS ≤ 4) compared to those with BM. This is consistent with a previous meta-analysis that indicated a high mortality rate associated with TBM, estimated to be as high as 41% among patients with meningitis [27], while reporting a low mortality associated with BM around 0.5–1% with treatment [6, 28]. Long term neurological sequelae remain frequent in both clinical entities. The risk of neurological sequelae in TBM has been estimated through a meta-analysis to be as high as 28.7% [29] and in BM, the rate is about 20–30% [15].

Despite the difficulties encountered in attempts to differentiate between BM and TBM, it is important because the duration of therapy and types of regimens differ. Unlike TBM [30], it is not standard of practice to initiate empirical treatment for brucella in those suspected of having BM. Corticosteroids are an important part of the treatment in TBM [31] but it is infrequently used in specific neurobrucellar syndromes [32]. Moreover, medications such as rifampin, quinolones, or aminoglycosides, which are primary therapeutic options for treating brucellosis, are also employed as anti-tuberculosis medications. Errors in distinguishing between these two clinical entities may lead to the inadvertent use of monotherapy and inadequate treatment with anti-tuberculosis medications in a patient with TBM. This increases the risk of treatment failure and the development of resistance.

While the current study is one of the first to compare the clinical and paraclinical features of TBM and BM, it has several limitations. First, due to the retrospective identification of a considerable proportion of our patients, detailed clinical information was unavailable for all cases. Second, the sample size of this research was relatively low, resulting in a low-power status for our analysis. Additionally, this low-power state, in the case of a significant result, led to a wide range of interval estimation, making the findings inconclusive. Last but not least, the lack of long-term follow-up could influence our estimation of favorable versus unfavorable clinical outcomes for patients.

## Conclusions

In conclusion, differentiation between neurotuberculosis and neurobrucellosis in the endemic areas is important but challenging. There is a substantial overlap in the clinical, laboratory, and imaging features of these clinical entities. Although altered consciousness, hydrocephalus, meningeal enhancement, and ischemic foci on neuroimaging, and predominance of polymorphonuclear leukocytes in CSF analysis were more frequently present in patients with neurotuberculosis, these are not distinctive features and can be used only to weigh the two alternative hypothesis states. Clinicians should therefore be aware of these overlapping features to reduce the risk of misdiagnosis.

### Supplementary Information

**Supplementary Material 1.**

## Data Availability

The data underlying this article will be shared upon reasonable request to the corresponding author.

## Abbreviations

TBM: Tuberculous meningitis
BM: Brucella meningitis
CSF: Cerebrospinal fluid
STA: Standard tube agglutination
2-ME: 2-Mercaptoethanol
MRI: Magnetic resonance imaging
CT scan: Computed tomography scan
WBC: White blood cells
PACS: Picture archiving and communication system
GOS: Glascow outcome scale
RoGM: Ratio of geometric mean
PMN: Polymorphonuclear
SMD: Standardized mean difference
RR: Risk ratio
CI: Confidence interval
LOS: Length of hospital stay
TR: Time ratio
AIC: Akaike information criterion
BIC: Bayesian information criterion
mNGS: Metagenomic next-generation sequencing
ADEM: Acute disseminated encephalomyelitis
