# Supplementary material for: Chronic meningitis in adults: a comparison between neurotuberculosis and neurobrucellosis
Source: BMC Infect Dis. 2024 Apr 25;24:441. doi: 10.1186/s12879-024-09345-6 (PMC11046744; doi:10.1186/s12879-024-09345-6)
Supplement: Supplementary file 1 — Supplementary Material 1. [file 12879_2024_9345_MOESM1_ESM.docx]

**Supplemental Table 1** Evaluation of the information criterion for various survival parametric models to compare the median length of hospital stay between TBM and BM

| Type of distribution | AIC | BIC |
| --- | --- | --- |
| Exponential | 173.1 | 177.6 |
| Weibull | 157.1 | 163.8 |
| Log-normal | 148.7 | 155.4 |
| Log-logistic | **148.1** | **154.8** |

TBM, tuberculous meningitis; BM, brucella meningitis; AIC, Akaike information criterion; BIC, Bayesian information criterion
